# Corrigendum

**DOI:** 10.1111/pbi.13418

**Published:** 2020-06-12

The authors of Chen *et al. *(2019) have misquoted Nayar *et al. *(2013) in regard to CKX2, a mistake for which they apologize. To rectify this, the following changes should be made to their article:

In Table 2, the reference to Nayar *et al. *(2013) should be removed. The respective row in the table should read as follows:

**Table:** 

*CKX2* paper	Inadequate/misleading cytokinin measurements	Joshi *et al.* (2018)

In the first paragraph of page 622, the sentence ‘Two papers on CKX2 in rice give cause for concern.’ should read ‘Two papers on cytokinin in rice give cause for concern.’
